# Volume, patterns, and types of sedentary behavior and cardio-metabolic health in children and adolescents: a cross-sectional study

**DOI:** 10.1186/1471-2458-11-274

**Published:** 2011-05-04

**Authors:** Valerie Carson, Ian Janssen

**Affiliations:** 1 School of Kinesiology and Health Studies, Queen's University, Kingston, ON, Canada; 2 Department of Community Health and Epidemiology, Queen's University, Kingston, ON, Canada

## Abstract

**Background:**

Cardio-metabolic risk factors are becoming more prevalent in children and adolescents. A lack of moderate-to-vigorous intensity physical activity (MVPA) is an established determinant of cardio-metabolic risk factors in children and adolescents. Less is known about the relationship between sedentary behavior and cardio-metabolic health. Therefore, the objective was to examine the independent associations between volume, patterns, and types of sedentary behavior with cardio-metabolic risk factors among children and adolescents.

**Methods:**

The results are based on 2527 children and adolescents (6-19 years old) from the 2003/04 and 2005/06 National Health and Nutrition Examination Surveys (NHANES). A cardio-metabolic risk score (CRS) was calculated based on age- and sex-adjusted waist circumference, systolic blood pressure, non-high-density lipoprotein cholesterol, and C-reactive protein values. Volume and patterns of sedentary behavior and moderate-to-vigorous physical activity (MVPA) were measured objectively using accelerometers. Types of sedentary behavior were measured by questionnaire. A series of logistic regression models were used to examine associations.

**Results:**

Volume and patterns of sedentary behavior were not predictors of high CRS after adjusting for MVPA and other confounders (P > 0.1). For types of sedentary behavior, high TV use, but not high computer use, was a predictor of high CRS after adjustment for MVPA and other confounders. Children and adolescents who watched ≥4 hours per day of TV were 2.53 (95% confidence interval: 1.45-4.42) times more likely to have high CRS than those who watched <1 hour per day. MVPA predicted high CRS after adjusting for all sedentary behavior measures and other confounders. After adjustment for waist circumference, MVPA also predicted high non-obesity CRS; however, the same relationship was not seen with TV use.

**Conclusion:**

No association was observed between overall volume and patterns of sedentary behavior with cardio-metabolic risk factors in this large sample of children and adolescents. Conversely, high TV use and low MVPA were independently associated with cardio-metabolic risk factors. However, the association between high TV use and clustered cardio-metabolic risk factors appears to be mediated or confounded by obesity. Thus, TV and MVPA appear to be two separate behaviors that need to be targeted with different interventions and policies.

## Background

Cardio-metabolic risk factors, such as obesity, hypertension, dyslipidemia, and glucose intolerance, are known predictors of coronary heart disease and type 2 diabetes among adults [1]. These risk factors are becoming more prevalent in children and adolescents, and approximately 50% of American youth have at least one cardio-metabolic risk factor [2]. This is concerning as cardio-metabolic risk factors track from childhood to adulthood [1]. In addition, cardio-metabolic risk factors during adolescence predict the development of sub-clinical cardiovascular disease [3], coronary heart disease [4], and mortality in adulthood [5]. Therefore, improving the cardio-metabolic risk factor profile of young people has long-term implications on population health.

Physical inactivity is an established determinant of cardio-metabolic risk factors in children and adolescents [6]. The majority of physical inactivity research has focused on how inadequate moderate-to-vigorous intensity physical activity (MVPA) influences health [7]. However, even within highly active persons, MVPA accounts for only a fraction of total energy expenditure [8]. An emerging area of study is the relation between sedentary behavior and health [7]. Sedentary behavior refers to activities that involve minimal body movement and low energy expenditure [9]. To date, two studies have examined the relationship between the overall volume of sedentary behavior with a summary cardio-metabolic risk score among children and adolescents [10,11]. Although both studies found significant associations, the results need to be interpreted with caution. Specifically, neither study adjusted for MVPA even though MVPA is an independent predictor of cardio-metabolic risk factors [12].

In addition to the overall volume, the type of sedentary behavior appears to impact cardio-metabolic health. For example, a recent review reported that TV use is more strongly related to obesity than video game and computer use in young people [13]. A limitation of the existing screen time (i.e., TV, computer, video game) literature is that only one study has considered the impact of the overall volume of sedentary behavior on the observed relationships [14].

Along with volume and types of sedentary behavior, consideration should be given to the patterns in which sedentary behavior is accumulated. Prolonged bouts of sedentary behavior are associated with cardio-metabolic health in adults [15,16]. One study among adults reported that an increased number of breaks in sedentary behavior are associated with an improved cardio-metabolic risk factor profile, independent of total sedentary behavior time and MVPA [17]. No study has examined the relationship between patterns of sedentary behavior and cardio-metabolic risk factors in the pediatric population. Children and adolescents spend extended periods of time being sedentary in and outside of school [9,18,19] and have sporadic MVPA patterns [20]. It is unknown whether patterns of sedentary behavior independently impact cardio-metabolic risk factors in young people.

The purpose of this study was to comprehensively examine the relationships between the volume, patterns, and types of sedentary behavior with cardio-metabolic risk factors in children and adolescents. The specific objectives were to: (1) determine whether the volume of sedentary behavior predicts cardio-metabolic risk factors independent of MVPA; (2) determine whether patterns (bouts and breaks in bouts) of sedentary behavior predict cardio-metabolic risk factors independent of MVPA and the volume of sedentary behavior; and (3) determine whether different types of sedentary behavior are related to cardio-metabolic risk factors in a similar manner.

## Methods

### Participants

The study is based on the 2003-2004 and 2005-2006 cycles of the Nutrition Health and Nutrition Examination Survey (NHANES), a nationally representative cross-sectional survey of the US. NHANES consisted of a home interview and a physical exam conducted in a mobile examination center. Consent was obtained from all participants and their parents/guardians if <18 years old. NHANES was approved by the National Center for Health Statistics. The analyses presented here were approved by the Health Sciences Research Ethics Board at Queen's University.

A total of 6553 NHANES participants aged 6-19 attended the mobile examination center. We excluded 3208 participants with incomplete accelerometer information (as explained below) and an additional 918 participants with incomplete information on the cardio-metabolic outcome and covariate measures, leaving a final sample of 2527. There were no significant differences in age or gender (*P *> 0.05) between the participants that were included or excluded from the final sample. However, slightly more Hispanic (5.4%) and slightly less non-Hispanic white (3.2%) and non-Hispanic black (2.4%) participants were included in the final sample (*P *< 0.01).

### Measurement of Sedentary Behavior and Physical Activity

Volume of sedentary behavior, patterns of sedentary behavior (bouts and breaks in bouts), and physical activity variables were created by the authors based on the raw accelerometry data provided in the NHANES dataset. The Actigraph AM-7124 accelerometer (Actigraph, Ft. Walton Beach, FL) was the device used in the NHANES study. These are uniaxial accelerometers that record average intensities in one minute intervals or epochs. Participants were asked to wear the accelerometer on their right hip for 7 consecutive days except when sleeping or when the accelerometer could get wet.

Data from the accelerometers was downloaded by NHANES survey collaborators and checked for outliers and unreasonable or biologically implausible values, which were removed. Reasonable ranges of values were determined by criteria published in the literature and expert judgment [21]. Further data reduction was completed by the authors. Initially, we removed days with incomplete information. A day was considered complete if it contained ≥10 hours of wear time [22], non-wear time was defined as a period of >20 minutes of zero counts [22]. The second data reduction step involved removing participants with an insufficient number of days with complete data. Only participants with ≥4 complete days, including one weekend day, were included. The inclusion of a weekend day is important as there are significant weekday and weekend differences in MVPA and sedentary behavior [23,24]. A 4-5 day accelerometer monitoring period has a test-retest reliability of 0.8 among children in grades 1-6 and 0.7 among adolescent in grades 7-12 [23].

Before we derived the sedentary behavior and physical activity variables from the raw accelerometry data, epoch cut-points were selected. There is currently substantial variation on the cut-points used to define sedentary behavior and different intensities of physical activity [25]. We selected a cut-point of <100 counts per minute to define sedentary behavior [18]. For MVPA, a regression equation developed by Freedson and colleagues, for 6-18 year olds, was used to estimate metabolic equivalents (METs) for each epoch value, based on the participant's age and the epoch count [26]. MVPA was defined as ≥4.0 METs, based on established precedence in the pediatric exercise literature [25,27]. Low intensity physical activity was defined as epoch values between 100 counts and an equivalent of 4.0 METs.

**Figure 1:**
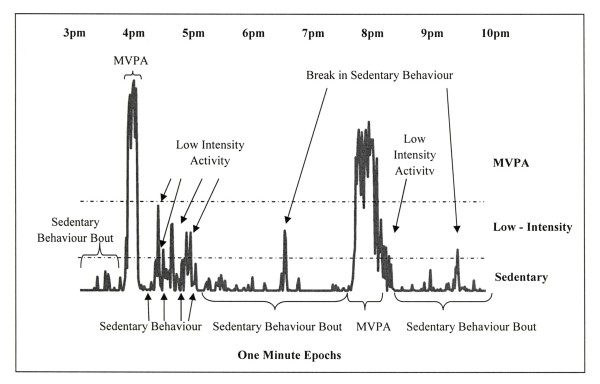
**Examples of physical activity and sedentary behavior variables derived from accelerometry data in one participant**. The x-axis represents 7 hours of time, in 1 minute epoch values, of the accelerometry measurements. The height of the data on the y-axis represents the intensity of the epoch values, with higher data points equaling higher intensities.

The next step was to derive the sedentary behavior and physical activity variables for each complete day of monitoring as illustrated in Figure 1. We calculated the volume of sedentary behavior, low intensity physical activity, and MVPA for each participant and divided these values by total wear time. To be defined as a sedentary behavior bout, there had to be ≥30 minutes with ≥80% of minutes below the 100 counts cut-point. The bout stopped when <80% was below the 100 counts cut-point or when there were ≥5 consecutive minutes ≥100 counts (Figure 1). Total minutes spent in sedentary behavior bouts were then divided by total wear time. A minimum 30 minute bout period of sedentary behavior was chosen to represent a 30 minute TV program or class in school. Also, sensitivity analyses indicated that the 30 minute bout period had a better model fit with cardio-metabolic risk factors than shorter bout periods (i.e., 5 or 10 minutes). Within each bout of sedentary behavior, we calculated the number of "break" minutes as those minutes equal to low intensity activity or MVPA. A variable reflecting the percentage of sedentary behavior bout time spent in breaks was calculated.

Finally, the type of sedentary behavior (TV or computer) was obtained by questionnaire from the NHANES dataset. TV and computer were selected because they were available in the dataset and they make up a large proportion of sedentary behavior time. For example, American youth spent 6-8 hours/day sedentary [18] and 4.5 hours/day using a TV or computer [28]. Proxy respondents answered the questions for participants aged 6-11 and participants aged 12-19 responded themselves. Two questions were asked, "Over the past 30 days, on average about how many hours per day did (you/your child) sit and watch TV or video?" and "Over the past 30 days, on average how many hours per day did (you/your child) use a computer or play computer games?" There were 7 response options ranging from "none" to "5 hours or more". We collapsed these response options into 4 groups (<1 hour, 1 hour, 2-3 hours, ≥ 4 hours) based on frequency distributions.

### Cardio-Metabolic Risk Factors

Waist circumference, systolic blood pressure, non-high-density lipoprotein cholesterol (non-HDL cholesterol), and C-reactive protein were the cardio-metabolic risk factors studied. These risk factors were selected because they were available in the NHANES dataset and because they capture different aspects of cardio-metabolic risk. All measurements were taken by trained personnel at the mobile examination center visit. Triglycerides and fasting glucose were not examined as cardio-metabolic risk factors due to the unavailability of fasting values in participants under the age of 12 in the NHANES dataset.

Waist circumference was measured to the nearest 0.1 cm at the level of the iliac crest. Waist circumference is an effective measure of abdominal adiposity among children and adolescents [29] and is a better predictor of cardio-metabolic risk factors than the body mass index [30]. Blood pressure was measured manually four consecutive times on the right arm while seated. We calculated the average blood pressures. We calculated non-HDL cholesterol by subtracting HDL cholesterol from total cholesterol [31]. HDL cholesterol was measured using the direct HDL immunoassay method and total cholesterol was measured enzymatically in serum in a series of coupled reactions using cholesteryl ester hydrolase, cholesterol oxidase, and peroxidase. Non-HDL cholesterol was chosen as the lipid marker because it is an important indicator of cardiovascular disease and diabetes risk among children and adolescents that it is not reliant upon a fasting blood sample [32]. C-reactive protein was measured by latex enhanced nephelometry. C-reactive protein was chosen as the inflammatory marker because of its availability in the dataset, known impact on cardiovascular disease, and because it is not reliant upon a fasting blood sample [33,34].

Waist circumference and non-HDL cholesterol were not normally distributed so they were log transformed by the authors prior to analyses. Age-adjusted values were created by the authors for each of the cardio-metabolic risk factors because they change with normal growth and maturation [35]. Using forward stepwise regression, each of the cardio-metabolic risk factors were regressed up to a full cubic polynomial in age (age, age^2^, age^3^) separately within males and females. Variables were allowed to enter or leave the model at *P *< 0.10. The standardized residuals were retained and used to represent the age-adjusted values. Participants were then ranked based on the residual for each risk factor. A mean of the ranks was used to represent a summary cardio-metabolic risk score (CRS). Blood pressure was not measured in children <8 years old, so the CRS for 6-7 year olds was limited to three risk factors. We categorized CRS into quartiles; the highest quartile denotes high CRS.

### Covariates

Age, gender, ethnicity (non-Hispanic white, non-Hispanic black, Hispanic, other), socioeconomic status (SES), smoking status, and diet were considered as covariates. The poverty-to-income ratio, provided within the NHANES dataset, is a ratio between family income and poverty threshold and was used to measure SES [36]. Smoking was assessed in NHANES by asking participants 12 and older, "Have you ever tried cigarette smoking, even 1 or 2 puffs?" We grouped participants into "yes" or "no" categories. Participants <12 years old were placed into the "no" category. Diet was assessed in NHANES via a 24 hour recall. We created four binary variables from the total nutrient values provided in the database: total fat (≤35% or >35% total calories), saturated fat (≤10% or >10% total calories), dietary cholesterol (≤300 or >300 mg/day), and sodium (≤2300 or >2300 mg/day) [37].

### Statistical Analysis

Analyses were completing using SAS version 9.2 (SAS Institute Inc., Cary, NC) and accounted for the complex design and sample weights of NHANES. Descriptive statistics were calculated. Relations between activity and sedentary behavior variables were determined using Pearson (continuous variables) and Spearman (categorical variables) correlations. Multiple logistic regression models were used to address the study objectives. All regression models predicted the highest CRS quartile and adjusted for various confounders including age, gender, ethnicity, SES, smoking, total fat, saturated fat, dietary cholesterol, sodium, and MVPA. To address objective 1 (volume of sedentary behavior), an initial regression model was run that included the sedentary behavior volume measure and all confounders except for MVPA. A second model was run that also adjusted for MVPA. To address objective 2 (patterns of sedentary behavior), initial regression models were run that included (a) the bouts of sedentary behavior measure and all confounders except MVPA, and (b) the breaks in bouts of sedentary behavior measure, volume of sedentary behavior, and all confounders except MVPA. Additional models were run that also adjusted for MVPA. To address objective 3 (types of sedentary behavior), initial regression models were run that included (a) TV use and all confounders except MVPA, and (b) computer use and all confounders except MVPA. Additional models were run that also adjusted for MVPA. Along with the analyses performed to address the main objectives, some supplemental analysis was conducted. First, age and gender interactions were explored in the primary analyses as well as sedentary behavior and MVPA interactions. Second, the relationship between MVPA and CRS was considered by running an initial logistic regression model that included the MVPA measure and all confounders. An additional model was run that also adjusted for overall volume of sedentary behavior. Third, the role of obesity as a mediator or confounder in the relationship between the sedentary behavior and MVPA variables with CRS was examined. Consistent with previous literature, a non-obesity CRS variable was created by removing waist circumference, and the above analyses were repeated predicting high non-obesity CRS, while further adjusting for waist circumference [10,12,38]. Finally, the relationship between the sedentary behavior and MVPA variables with individual CRS components was explored. All logistic regression models predicted the highest quartiles of the individual components. The same modeling strategies described above in this paragraph for the CRS outcome variable were used to explore these relationships.

## Results

### Descriptive Analyses

Participant characteristics are in Table 1. Approximately 49% were female and the median age was 13. Overall, the median values for the breakdown of the accelerometry wear time were 50.8% for sedentary behavior, 43.8% for low intensity activity, and 4.1% for MVPA. The median values for bouts of sedentary behavior lasting ≥30 minutes was 24.5%, with 13.5% of that time (or 0.03% of total wear time) spent in breaks. The average length of sedentary bouts was 64.5 minutes. Correlations between sedentary behavior and activity variables are in Table 2.

**Table 1:** Participant characteristics

Variables	Total (*N = 2527*)
Age (years)	13 (10-16)
Gender (%)	
Male	50.8
Female	49.2
Race (%)	
Non-Hispanic white	38.3
Non-Hispanic black	24.4
Hispanic	32.7
Other	4.6
Accelerometer-derived variables	
Total wear time (minutes/day)	834 (779-894)
Sedentary behavior (% of total time)	50.8 (42.1-58.9)
Bouts of sedentary behavior (% of total time)	24.5 (14.4-36.1)
Breaks in bouts of sedentary behavior (% of bouts)	13.5 (12.1-15.3)
MVPA (% of total time)	4.1 (1.6-8.5)
Low intensity activity (% of total time)	43.8 (38.0-49.6)
Questionnaire-derived variables	
TV (hours/day)	2.0 (1.0-3.0)
Computer (hours/day)	0.5 (0.0-1.0)
Cardio-metabolic risk score components	
Waist circumference (cm)	72.9 (64.5-82.7)
Systolic blood pressure (mmHg, N = 2265)	107 (100-114)
Non-HDL (mmol/l)	2.7 (2.3-3.2)
C - reactive protein (mmol/l)	0.002 (0.001-0.007)

Data presented as median (inter-quartile range) or %. MVPA = moderate-to-vigorous intensity physical activity; Non-HDL = non-HDL cholesterol

**Table 2:** Correlations between sedentary behavior and physical activity variables.

	Volume of SB	Bouts of SB	Breaks in Bouts of SB	TV	Computer	MVPA	Low Intensity PA
Volume of SB	-	.84	-.20	.08	.19	-.70	-.90
Bouts of SB		-	-.26	.05	.21	-.52	-.80
Breaks in Bouts of SB			-	-.02	-.11	-.03	-.77
TV				-	.15	-.06	-.07
Computer					-	-.15	-.18
MVPA						-	.31
Low Intensity PA							-

SB = sedentary behavior; MVPA = moderate-to-vigorous intensity physical activity; PA = physical activity.

All correlations were significant (P ≤ 0.05) except for the correlations between Breaks in Bouts of SB with MVPA and TV

### Objective 1: Volume of Sedentary Behavior

The prevalence of high CRS did not differ according to the volume of sedentary behavior (Table 3, P_trend _= 0.15). The volume of sedentary behavior did not predict (P_trend _≥ 0.2) high CRS after adjusting for various confounders (age, gender, race, SES, smoking, total fat, saturated fat, dietary cholesterol, sodium; model 1) and MVPA (model 2).

**Table 3:** High CRS according to volume, patterns, and types of sedentary behavior.

	Prevalence	Model 1	Model 2
	Total (*N = 2527*)	OR (95% CI)	OR (95% CI)
*Volume of sedentary behavior*			
Quartile 1	23.0	1.00	1.00
Quartile 2	24.1	0.79 (0.48-1.31)	0.77 (0.46-1.29)
Quartile 3	27.7	0.90 (0.53-1.53)	1.13 (0.64-2.01)
Quartile 4	25.4	0.87 (0.48-1.55)	0.76 (0.42-1.37)
	P _trend _= 0.15	P_trend _= 0.20	P_trend _= 0.68
*Bouts of sedentary behavior*			
Quartile 1	22.7	1.00	1.00
Quartile 2	24.8	1.15 (0.72-1.85)	0.96 (0.58-1.57)
Quartile 3	28.1	1.43 (0.84-2.45)	0.99 (0.58-1.73)
Quartile 4	24.6	1.31 (0.74-2.32)	0.98 (0.55-1.74)
	P_trend _= 0.24	P_trend _= 0.27	P_trend_= 0.80
*Breaks in bouts of sedentary behavior *			
Quartile 1	23.3	1.00	1.00
Quartile 2	26.3	1.20 (0.74-1.94)	1.21 (0.75-1.95)
Quartile 3	26.9	0.81 (0.50-1.32)	0.78 (0.47-1.28)
Quartile 4	23.8	1.00 (0.60-1.66)	1.03 (0.62-1.70)
	P_trend_= 0.78	P_trend _= 0.66	P_trend _= 0.67
*TV*			
<1 hour	19.8	1.00	1.00
1 hour	23.8	1.06 (0.58-1.92)	1.15 (0.64-2.04)
2-3 hours	22.8	1.59 (0.94-2.70)	1.63 (0.96-2.74)
≥4 hours	33.2	2.57 (1.45-4.56)*	2.53 (1.45-4.42)*
	P_trend _<0.01	P_trend_<0.01	P_trend_<0.01
*Computer*			
<1 hour	25.8	1.00	1.00
1 hour	22.2	1.21 (0.80-1.82)	1.17 (0.77-1.77)
2-3 hours	27.0	1.27 (0.82-1.96)	1.21 (0.79-1.87)
≥4 hours	23.7	0.69 (0.34-1.38)	0.56 (0.27-1.12)
	P_trend _= 0.83	P_trend _= 0.76	P_trend _= 0.75

High CRS refers to the highest quartile of CRS. Model 1: adjusted for age, gender, race, SES, smoking, total fat, saturated fat, cholesterol, and sodium. Model 2: Adjusted for covariates in Model 1 and MVPA. For the breaks in sedentary behavior variable, Model 1 and 2 were also adjusted for the volume of sedentary behavior. *P ≤ 0.05.

### Objective 2: Patterns of Sedentary Behavior

The prevalence of high CRS did not vary across quartiles of the bouts of sedentary behavior and the breaks in bouts of sedentary behavior measures (Table 3, P_trend _> 0.2). The two sedentary behavior pattern variables did not predict (P_trend _> 0.2) high CRS after adjusting for various confounders, the volume of sedentary behavior, (model 1) and MVPA (model 2).

### Objective 3: Types of Sedentary Behavior

The prevalence of high CRS increased significantly with increasing hours of TV use (Table 3, P_trend _< 0.01). High TV use remained a significant predictor of high CRS after adjustment for various confounders (model 1) and MVPA (model 2). Participants who watched ≥4 hours/day of TV were 2.53 (95% confidence interval (CI): 1.45-4.42) times more likely to have high CRS than those who watched <1 hour/day. Computer use was not related to high CRS (P_trend _> 0.7).

### Additional Analyses

There were no significant gender and age interactions in any of the models that examined the relationship between the volume, patterns, and types of sedentary behavior with high CRS. As well, there were no significant sedentary behavior and MVPA interactions.

After adjusting for confounders and the volume of sedentary behavior, participants in the second (odds ratio = 0.44, 95% CI: 0.27-0.71), third (0.30, 0.16-0.55), and fourth (0.16, 0.06-0.40) MVPA quartiles were significantly less likely to have high CRS than participants in quartile one (P_trend _< 0.01). Similar associations were observed when predicting high non-obesity CRS after further adjusting for waist circumference. However, TV use was not associated with high non-obesity CRS. For example, participants who watch ≥4 hours/day of TV were not significantly (1.09, 0.63-1.86) more likely to have high non-obesity CRS than those who watched <1 hour/day after further adjusting for waist circumference.

The observed associations between the sedentary behavior and MVPA variables with the individual CRS components (high waist circumference, systolic blood pressure, non-HDL cholesterol, and C-reactive protein) were similar to the results of the primary CRS analyses. For example, overall volume and patterns of sedentary behavior as well as computer use were not associated with any of the individual CRS components, after adjusting for various confounders (age, gender, race, SES, smoking, total fat, saturated fat, dietary cholesterol, sodium) and MVPA (Table 4). Conversely, MVPA was associated with all four individual components of CRS in a dose-response manner, after adjustment for various confounders and sedentary behavior (P_trend _≤ 0.05; data not shown). We also found that the odds of high waist circumference, non-HDL cholesterol, and C-reactive protein increased in a dose-response manner with increasing TV use (P_trend _≤ 0.05; Table 4).

**Table 4:** High waist circumference, systolic blood pressure, non-HDL, and C-reactive protein according to volume, patterns, and types of sedentary behavior.

	Waist Circumference	Systolic Blood Pressure	Non-HDL	C-Reactive Protein
	OR (95% CI)	OR (95% CI)	OR (95% CI)	OR (95% CI)
*Volume of sedentary behavior*				
Quartile 1	1.00	1.00	1.00	1.00
Quartile 2	1.05 (0.63-1.76)	0.83 (0.48-1.44)	0.99 (0.60-1.60)	0.84 (0.50-1.40)
Quartile 3	1.06 (0.61-1.83)	1.04 (0.57-1.90)	1.53 (0.88-2.64)	1.04 (0.60-1.78)
Quartile 4	0.86 (0.46-1.60)	0.83 (0.46-1.50)	1.35 (0.76-2.40)	1.01 (0.54-1.88)
	P _trend _= 0.66	P_trend _= 0.72	P_trend _= 0.16	P_trend _= 0.81
*Bouts of sedentary behavior*				
Quartile 1	1.00	1.00	1.00	1.00
Quartile 2	1.24 (0.76-2.02)	0.96 (0.56-1.66)	1.41 (0.88-2.26)	0.88 (0.54-1.42)
Quartile 3	0.89 (0.54-1.47)	0.76 (0.42-1.38)	1.43 (0.85-2.42)	1.20 (0.68-1.77)
Quartile 4	0.88 (0.49-1.58)	0.92 (0.50-1.71)	1.54 (0.87-2.71)	1.04 (0.59-1.85)
	P_trend _= 0.42	P_trend _= 0.62	P_trend _= 0.16	P_trend _= 0.69
*Breaks in bouts of sedentary behavior *				
Quartile 1	1.00	1.00	1.00	1.00
Quartile 2	1.31 (0.82-2.08)	0.91 (0.55-1.49)	1.06 (0.67-1.67)	1.41 (0.88-2.27)
Quartile 3	0.88 (0.56-1.36)	0.78 (0.46-1.33)	0.81 (0.51-1.29)	1.28 (0.79-2.09)
Quartile 4	1.06 (0.66-1.70)	1.12 (0.66-1.89)	0.98 (0.61-1.56)	1.17 (0.72-1.88)
	P_trend _= 0.77	P_trend _= 0.77	P_trend _= 0.69	P_trend _= 0.69
*TV*				
<1 hour	1.00	1.00	1.00	1.00
1 hour	0.85 (0.46-1.55)	1.16 (0.62-2.15)	0.92 (0.52-1.62)	1.11 (0.61-2.01)
2-3 hours	1.59 (0.93-2.71)	1.21 (0.71-2.06)	1.41 (0.89-2.32)	1.14 (0.68-1.91)
≥4 hours	2.35 (1.29-4.27)*	1.30 (0.71-2.38)	1.54 (0.89-2.68)	1.75 (0.99-3.11)
	P_trend_< 0.01	P_trend _= 0.41	P_trend _= 0.03	P_trend _= 0.05
*Computer*				
<1 hour	1.00	1.00	1.00	1.00
1 hour	1.26 (0.84-1.89)	0.79 (0.50-1.26)	1.34 (0.91-1.97)	1.33 (0.90-1.97)
2-3 hours	1.23 (0.78-1.94)	1.13 (0.69-1.84)	0.97 (0.63-1.49)	1.26 (0.81-1.95)
≥4 hours	0.88 (0.44-1.76)	0.81 (0.37-1.74)	1.14 (0.59-2.20)	0.76 (0.39-1.50)
	P_trend _= 0.58	P_trend _= 0.94	P_trend _= 0.73	P_trend _= 0.62

Non-HDL = non-HDL cholesterol. High waist circumference, systolic blood pressure, non-HDL, and C-reactive protein refers to the highest quartile of these variables. All models are adjusted for age, gender, race, SES, smoking, total fat, saturated fat, cholesterol, sodium, and MVPA. The model for breaks in sedentary behavior is also adjusted for the volume of sedentary behavior. *P ≤ 0.05.

Finally, the results of all of the aforementioned analyses were consistent when the analyses were repeated using linear regression with continuous CRS and continuous individual CRS component outcome variables.

## Discussion

This study examined associations between the volume, patterns, and types of sedentary behavior with cardio-metabolic risk factors in 6-19 year olds. Although this representative sample spent 50.8% of their waking hours in sedentary behavior, the volume of sedentary behavior was not an independent predictor of high-risk cardio-metabolic factor values. Similarly, patterns of sedentary behavior, such as the amount of time in bouts of sedentary behavior ≥30 minutes, was not related to cardio-metabolic risk factors. However, the type of sedentary behavior was important. More specifically, the amount of time spent watching TV was related to cardio-metabolic risk factors, while computer use was not.

Among adults, the volume of sedentary behavior, as measured objectively by accelerometers, is associated with a clustering of cardio-metabolic risk factors [39], waist circumference [39], and glucose intolerance [40] that are independent of MVPA and other confounders. The relationship between the volume of sedentary behavior and cardio-metabolic risk factors appears to be stronger and more consistent in adults than young people. In our study, the volume of objectively measured sedentary behavior was not associated with high CRS or its individual components. Two previous cross-sectional studies within children and/or adolescents have examined the association between the volume of sedentary behavior, measured by an accelerometer, with a summary cardio-metabolic risk score [10,11]. Positive associations were observed in both studies. However, neither study examined whether these associations were independent of MVPA, which is an important limitation, given that MVPA is related to sedentary behavior (see Table 2). In addition, five previous studies within children and adolescents, all cross-sectional in design, have examined the association between the volume of sedentary behavior, measured by an accelerometer, with individual risk factors such as insulin resistance [41], blood pressure [14], and various measures of obesity [42-44]. One of these studies found moderate positive associations (*r = *0.21) between the volume of sedentary behavior and insulin resistance among 9-10 year old children independent of obesity [41]. Similarly, this study did not determine whether the associations were independent of MVPA. In the three studies that examined obesity measures, associations with volume of sedentary behavior did not exist or was severely attenuated after adjustment for MVPA [42-44].

Emerging evidence in adults suggests that patterns in which sedentary behavior is accumulated may independently impact cardio-metabolic risk. More specifically, a cross-sectional study of 168 Australian adults found that the frequency of breaks in sedentary behavior was negatively related to waist circumference, triglycerides, and glucose levels, independent of MVPA and the overall volume of sedentary behavior [17]. We are unaware of previous studies that have examined these associations in children or adolescents. Thus, our observation that patterns of sedentary behavior, including sedentary behavior bouts and breaks in bouts of sedentary behavior, were not related to cardio-metabolic risk factors in 6-19 year olds makes a novel contribution to the literature. It is possible that the differences in results between the present study and the previously mentioned adult study [17] is explained by a physiological difference in the way sedentary behavior impacts health in adults and young people. It is also possible that the different results are due to differences in the way "breaks" were measured in the two studies. While the present study looked at the frequency of breaks within prolonged (≥30 minutes) bouts, the Australian study counted a break any time the participant moved from a sedentary minute to a minute above the 100 count per minute accelerometry threshold [17]. Due to the dearth of information, more research is needed to better understand the relationship between patterns of sedentary behavior and cardio-metabolic health in all ages.

Our third objective was to determine if different types of sedentary behavior impact cardio-metabolic health in a similar manner. Several studies among young people have found associations between TV and total screen time with individual cardio-metabolic risk factors and the metabolic syndrome [12,14,45,46]. To our knowledge only one of these studies reported on the impact of different screen time measures on cardio-metabolic risk factors other than obesity [14]. This particular study found that blood pressure was associated with TV but not with computer use [14]. Also, a recent literature review found that TV use is more strongly associated with obesity in children and adolescents than is computer use [13]. Likewise, we found that the odds of a high CRS increased in a dose-response manner within increasing TV volume, independent of MVPA, but that computer use did not predict CRS. Similar associations were seen with the individual components of CRS. There are two possible explanations for these findings. First, amongst the sedentary behaviors, TV may be at the lowest end of the energy expenditure spectrum. In fact, one study reported that energy expenditure in children and adolescents was lower while watching TV then while sleeping [47]. Second, TV encourages between meal snacking [48] and is associated with a greater exposure to junk food advertisements than other screen time measures [49]. Even though various dietary measures (total fat, saturated fat, cholesterol, and sodium) were adjusted for in this study, residual confounding may have been present. Future research needs to consider the impact of other types of sedentary behavior (reading, homework, etc.) on the health of young people.

Interestingly, in the present study the CRS variable was predicted by a self-report measure of TV use but not an objective measure of overall sedentary behavior volume or the overall volume of sedentary behavior accumulated in prolonged bouts. Also, TV use was poorly correlated (r ≤ 0.08) to these two objective measures. There are three possible explanations for these observations. First, the uniaxial accelerometer used in NHANES may not be sensitive enough to differentiate between sitting and standing like an inclinometer [50]. Also, participants may have been more likely to keep their accelerometer on during their daily activities and MVPA, and take it off later in the evening while watching TV [51]. Therefore, the objectively measured sedentary behavior may not have captured 100% of the sedentary behavior for some participants. Second, the specific sedentary behavior of TV may have a unique impact on cardio-metabolic risk factors due to its impact on energy expenditure and intake, as previously discussed. Third, the catchment period of sedentary behavior differed between the self- report measure (past 30 days) and the accelerometer measure (7 days). Perhaps, the longer catchment period better reflects typical behavior compared to the shorter period.

We also examined the association between objectively measured MVPA with CRS and non-obesity CRS. The finding that MVPA was strongly and independently associated with cardio-metabolic risk factors in a dose-response manner is consistent with previous literature [52]. For example, a dose-response relationship between MVPA and cardio-metabolic risk factors was observed in approximately 2000 participants of the European Youth Heart Study [6]. As with the present study, the associations between MVPA and clustered cardio-metabolic risk factors within children and adolescents have been reported to be independent of TV use and obesity [12]. However, similar to the present study, the association between TV use and clustered cardio-metabolic risk factors do not appear to be independent of obesity [12]. This suggests that obesity mediates or confounds the relationship between TV use and clustered cardio-metabolic risk factors [12].

Strengths of this study include the objective measures of MVPA and most of the sedentary behavior variables as well as our novel approach used to examine patterns of sedentary behavior. Limitations of the study include the cross-sectional design, which limits the ability to make causal inferences about the relationships. Also, our final sample was not representative of the population in terms of ethnicity. In addition, the accelerometers used may not be sensitive enough to differentiate between sitting and standing [50]. Furthermore, we only considered two types of sedentary behavior, both of which were measured via self-report. The biases with these self-reported measures may have results in an underestimation of the strength of associations between the TV, computer, and CRS variables. Finally, although a variety of confounders were considered, we were not able adjust for pubertal development, a factor which influences physiological processes [53].

## Conclusions

No association was observed between overall volume and patterns of sedentary behavior with cardio-metabolic risk factors in this large sample of children and adolescents. Conversely, TV use and low MVPA were both independently associated with cardio-metabolic risk factors. However, the association between high TV use and clustered cardio-metabolic risk factors appears to be mediated or confounded by obesity. In addition, the TV and MVPA variables were poorly correlated with one another. This suggests that these are two separate behaviors, and that different policy and intervention programs are needed to increase MVPA and decrease TV use in an effort to prevent and reduce cardio-metabolic risk factors.

## Competing interests

The authors declare that they have no competing interests.

## Authors' contributions

VC assisted with the design of the study, led the statistical analysis, and wrote the initial draft of the article. IJ assisted with the design of the study, provided insight and guidance on the statistical analysis, and revised the manuscript for important intellectual content. Both authors approve the version that has been submitted.

## Pre-publication history

The pre-publication history for this paper can be accessed here:

http://www.biomedcentral.com/1471-2458/11/274/prepub
